# Diverse protocols for measuring glomerular filtration rate using iohexol clearance

**DOI:** 10.1093/ndt/gfae006

**Published:** 2024-01-16

**Authors:** Abdulfataah A A Mohamed, Arend Bökenkamp, Etienne Cavalier, Pierre Delanaye, Natalie Ebert, Marco van Londen

**Affiliations:** Department of Internal Medicine, Division of Nephrology, University Medical Center Groningen, Groningen, The Netherlands; Department of Clinical Pharmacy and Pharmacology, University Medical Center Groningen, Groningen, The Netherlands; Department of Pediatric Nephrology, Emma Children's Hospital, Amsterdam University Medical Centers, Amsterdam, The Netherlands; Department of Clinical Chemistry, CHU de Liège, CIRM, University of Liége, Liège, Belgium; Nephrology-Dialysis-Transplantation, CHU Sart Tilman, University of Liége, Liége, Belgium; Service de Néphrologie-Dialyse-Aphérèse, Hôpital Universitaire Carémeau, Nîmes, France; Charité-Universitätsmedizin Berlin, Institute of Public Health, Berlin, Germany; Department of Internal Medicine, Division of Nephrology, University Medical Center Groningen, Groningen, The Netherlands

To the Editor,

Accurate measurement of kidney function is necessary in the diagnosis and monitoring of patients with kidney disease [1]. The Kidney Disease: Improving Global Outcomes (KDIGO) study group recommends the use of exogenous filtration markers to measure glomerular filtration rate (GFR) in situations where accurate assessment of kidney function is important and estimated GFR is not sufficient [2]. Among the various exogenous markers available, iohexol is often used due to its favourable pharmacokinetic properties, availability, feasibility, safety profile and established validity in measuring GFR [3]. While iohexol-based GFR measurement has demonstrated its accuracy, the lack of standardization in the protocols utilized across different centres poses a major challenge [3, 4]. While standardization is vital to ensure that measurements obtained from different centres are comparable, the differences between iohexol-based protocols are not known. In this study, we aimed to identify differences between iohexol plasma clearance–based measured GFR (mGFR) protocols in different hospitals throughout Europe and North America (the USA and Canada).

We conducted a standardized online survey among clinical chemists and nephrologists from Europe and North America. The survey was distributed to all contacts of the members of the European Kidney Function Consortium (EKFC) that use iohexol clearance measurements in clinical practice or research. The survey was composed of 23 questions covering pre-analytical, analytical and post-analytical topics. The questions on pre-analytical topics concerned referral and indication, pre-measurement instructions, iohexol administration, blood sampling and handling. The analytical phase concerned methodology of iohexol measurement, type of detector [ultraviolet (UV) or mass spectrometer (MS)], type of liquid chromatography (LC) and external quality assessment (EQA). The post-analytical phase concerned the reporting and interpretation of results. The survey was sent by e-mail (through Google Forms) to 18 hospitals in Europe and North America. The data were collected between December 2019 and April 2022. Data were analysed using descriptive statistics and converted into graphs using Excel (Microsoft, Redmond, WA, USA). Results from the survey are displayed as number and percentage.

A total of 15 of the 18 addressed physicians answered the survey. Replies to the survey came from hospitals in Europe [*n* = 13 (87%)] or North America [*n* = 2 (13%)]. Among the 15 respondents, referrals for mGFR were ordered by the nephrology department [*n* = 6 (40%)], other departments [*n* = 5 (33%)] or researchers [*n* = 4 (27%)]. Indications mentioned most often were screening for living kidney donation [*n* = 5 (33%)], drug dosing [*n* = 4 (27%)], abnormal body composition [*n* = 3 (20%)] and estimated GFR (eGFR) disagreement [*n* = 2 (13%)]. Other indications were CKD classification [*n* = 1 (7%)], transplantation [*n* = 1 (7%)], paediatric patients [*n* = 2 (13%)], risk assessment in renal failure [*n* = 2 (13%)] and acute kidney injury [*n* = 1 (7%)]. Most centres cited multiple indications, while four centres (27%) did not specify the indication.

Most centres gave pre-measurement instructions for patients [*n* = 11 (73%)]. As shown in Fig. 1A, pre-measurement instructions were specified as restrictions on diet, including fasting and lowering protein/carbohydrate intake [*n* = 8 (53%)], medication [*n* = 2 (13%)], caffeine [*n* = 5 (33%)], tobacco use [*n* = 1 (7%)] and ensuring sufficient fluid intake [*n* = 3 (20%)]. The kidney function measurement was performed in the morning [*n* = 10 (67%); Fig. 1B], mostly in the morning [*n* = 3 (20%)] or throughout the day [*n* = 2 (13%)].

**Figure 1:**
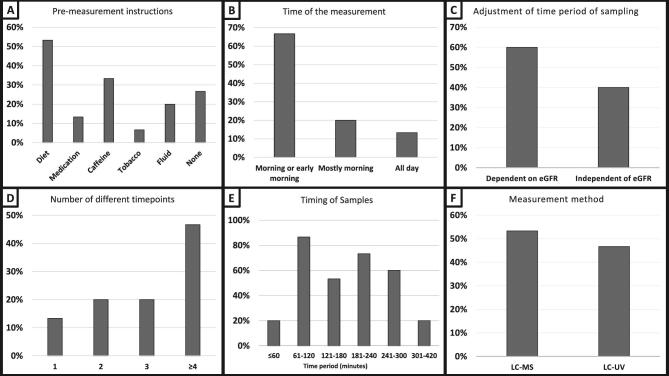
Survey details on iohexol-based mGFR protocols. The different methodologies utilized when measuring GFR using iohexol plasma clearance are based on a survey sent to 15 centres. **(A)** The pre-measurement instructions given to the patients. Centres could provide multiple answers. **(B)** Time of day at which the measurement was performed. **(C)** Adjustment of the time schedule for blood sampling, whether this was dependent on the expected GFR of the patient or not. **(D)** The total number of time points at which samples were collected. **(E)** Time period, after iohexol administration, in which samples were collected. **(F)** The measurement methods used to analyse iohexol.

Iohexol was given in a fixed dose of 5 ml (240 or 300 mg/ml) in most centres [*n* = 10 (67%)]. The remaining centres either calculated the dose based on weight [*n* = 2 (13%)], administered a fixed dose of 6 ml [*n* = 1 (7%)] or did not specify the administered dose [*n* = 2 (13%)]. The administered iohexol dose was verified by weighing the syringe before and after administration [gravimetric verification, *n* = 12 (80%)] or by volumetric verification [*n* = 2 (13%)]. The centres that reported on flushing with saline after iohexol administration [*n* = 8 (53%)], flush with at least 5 ml 0.9% sodium chloride. One centre performed no verification of the iohexol dose after administration [*n* = 1 (5%)]. Most centres used serum [*n* = 7 (47%)], lithium heparin [*n* = 4 (27%)], ethylenediaminetetraacetic acid [*n* = 2 (13%)] or sodium heparin [*n* = 1 (7%)] tubes for drawing blood samples; one centre did not specify the tubes used.

Fig. 1C shows that nine (60%) centres adjusted the period over which samples were collected depending on the expected GFR of the patient using eGFR. As shown in Fig. 1D, the total number of time points at which samples were collected is either one [single sample, *n* = 2 (13%)] or more than one [multiple samples, *n* = 13 (87%)]. The timing of sample collection after administration of iohexol occurs across different time periods (Fig. 1E): over a period of ≤60 min [*n* = 3 (20%)], 61–120 min [*n* = 13 (87%)], 121–180 min [*n* = 8 (53%)], 181–240 min [*n* = 11 (73%)], 241–300 min [*n* = 9 (60%)] or 301–420 min [*n* = 3 (20%)]. When asked about the timing of sample collection, centres could provide multiple answers. Most centres [*n* = 12 (80%)] drew one blood sample per time point. Blood tubes were centrifuged between 7 and 10 min in most centres [*n* = 11 (73%)].

Fig. 1F shows that iohexol was determined using either LC-MS [*n* = 8 (53%)] or LC-UV [*n* = 7 (47%)]. Centres used a one-compartment kinetic model [*n* = 8 (53%)], two-compartment model [*n* = 3 (20%)], a model dependent on the measurement type [*n* = 2 (13%)] or did not specify the model details [*n* = 2 (13%)]. Most centres [*n* = 10 (67%)] used the Bröchner–Mortensen method for correcting mGFR, while others use Ng correction [*n* = 1 (7%)], no correction [*n* = 1 (7%)] or did not specify correction [*n* = 3 (20%)]. For body surface area (BSA) adjustment, centres used the Du Bois and Du Bois formula [*n* = 6 (40%)], the Haycock and Schwarz formula [*n* = 2 (13%)], no adjustment [*n* = 1 (7%)] or did not specify adjustment [*n* = 6 (40%)].

EQA was performed for the iohexol kidney function test [*n* = 10 (67%)]. In most centres this was done by the EQUALIS laboratory (Uppsala, Sweden) [*n* = 7 (47%)]; other centres confirmed that EQA was performed but did not specify the EQA scheme [*n* = 3 (20%)]. The remaining centres reported no EQA [*n* = 3 (20%)] or did not specify whether EQA was performed [*n* = 2 (13%)].

This survey shows a large heterogeneity in pre-analytical, analytical and post-analytical protocols for iohexol-based mGFR. While iohexol-based mGFR has demonstrated its accuracy, this study highlights a lack of protocol standardization.

Patient instructions regarding protein intake and tobacco use, both associated with an increase in GFR, are important [5, 6]. The time of measurement influences results due to circadian variations in GFR [7]. The accuracy of measurements depends on the time points at which samples are drawn, which need to be adapted based on the expected GFR [8, 9]. Single-sample methods may be of equivalent quality to multisample methods, but the decision on the use of a single-sample method or multisample method should be made beforehand since it has implications regarding goodness of fit [3, 10]. The timing at which samples are taken, different in most survey participants, can lead to both systematic over- and underestimation of the mGFR [11]. Also, the compartment corrections and BSA adjustments varied between survey participants, while both influence results [12]. Most centres performed EQA for the measurement, which is crucial for reproducibility, particularly since there is no internationally standardized iohexol assay.

Limitations of our study are the sample size, limited to EKFC contacts, and the relatively large number of unspecified details. Also, we did not gather enough feedback on specific populations, such as paediatric patients. In summary, there is a need to standardize iohexol-based mGFR. This may improve clinical decision making and patient care and advance kidney function research.

## Supplementary Material

gfae006_Supplemental_File
