# Supplementary figures and images for: Diverse protocols for measuring glomerular filtration rate using iohexol clearance
Source: Nephrol Dial Transplant. 2024 Jan 16;39(6):1037–9. doi: 10.1093/ndt/gfae006 (PMC11210064; doi:10.1093/ndt/gfae006)

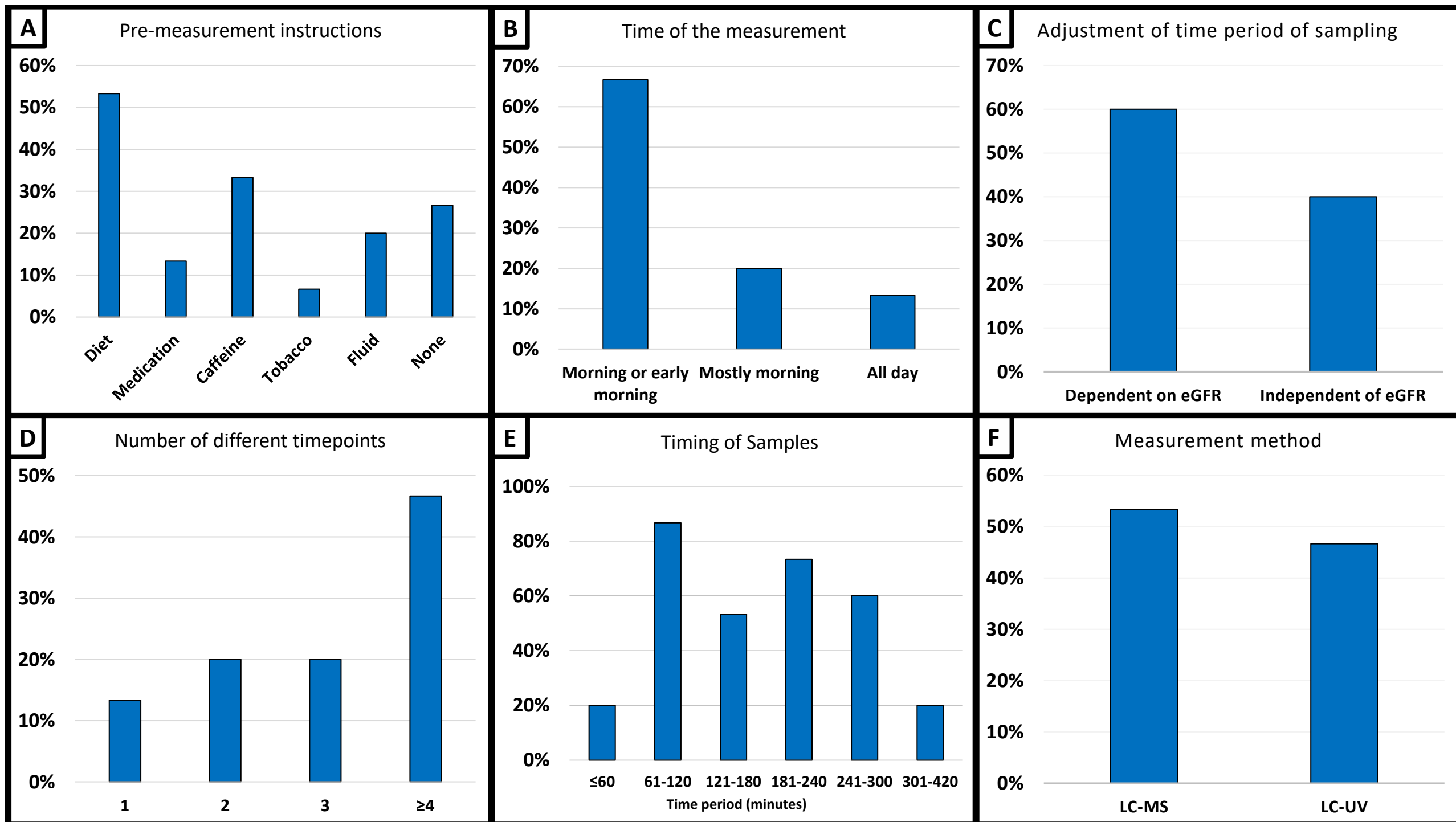

Supplement: gfae006_Supplemental_File [file gfae006_Supplemental_File.pdf]
